# YouTube as a source of information on retrograde ejaculation: insights into content reliability

**DOI:** 10.1038/s41443-025-01124-4

**Published:** 2025-07-02

**Authors:** Abdullah Akdağ, Erhan Ateş, Çağatay Özsoy

**Affiliations:** 1 Basaksehir Cam and Sakura City Hospital, Department of Urology, Istanbul, Türkiye; 2 Aydin Adnan Menderes University, Department of Urology, Aydin, Türkiye

**Keywords:** Sexual dysfunction, Urogenital reproductive disorders

## Abstract

This study evaluates the accuracy and reliability of YouTube videos on retrograde ejaculation, a condition affecting male fertility and quality of life. A systematic search conducted on December 11, 2024, identified 97 relevant videos from an initial pool of 545. Videos were analyzed using the DISCERN tool and Global Quality Score to assess reliability and educational quality. Reliable videos, comprising 54.6% of the sample, were significantly more often uploaded by universities, professional organizations, or nonprofit physician groups when compared to non-reliable videos (*P* = 0.006). When comparing different uploader subgroups, videos from universities, professional organizations, or nonprofit physician groups demonstrated significantly higher DISCERN and Global Quality Scores (*P* = 0.006, *P* < 0.001, respectively). In contrast, based on the overall distribution of uploader types among the entire video sample, videos uploaded by individuals or for-profit entities were more frequently classified as non-reliable (*P* = 0.005). Notably, viewership metrics did not differ significantly between reliable and nonreliable videos (*P* = 0.552), highlighting the challenge of discerning accurate content based on popularity alone. The findings underscore the importance of promoting high-quality, evidence-based information from credible sources on platforms like YouTube. Addressing misinformation and enhancing the visibility of reliable content are critical to improving patient education and decision-making in managing retrograde ejaculation.

## Introduction

Retrograde ejaculation (RE) is a rare but significant urological condition in which semen fails to exit through the urethra during ejaculation and instead redirects into the bladder, often caused by surgical procedures, neurologic disorders, or pharmacologic interventions, impacting male fertility and quality of life [1]. Despite its implications, patients with RE are often reluctant to discuss their symptoms, leading to delays in seeking professional medical advice. Factors contributing to this reluctance include embarrassment, lack of awareness, and the misconception that the condition will resolve on its own [2]. As a result, many patients remain misdiagnosed or untreated, further exacerbating the potential negative impact on their quality of life.

Individuals’ information needs vary depending on their specific health concerns. Beyond fulfilling the need for medical information, seeking health-related content on social media provides additional benefits, such as social and emotional support through peer-to-peer interactions. However, these advantages are often outweighed by concerns regarding the credibility and reliability of information, which can ultimately reduce engagement and trust in online health content [3]. In recent years, the internet has become a key source of health-related information, with platforms like YouTube serving as a widely used medium [4]. Because of its accessibility and multimedia format, YouTube attracts individuals seeking health information, including insights into less common conditions such as RE. However, since content on the platform is generated by a diverse range of creators, concerns arise regarding the accuracy, reliability, and completeness of the provided information. Research analyzing YouTube content on various medical topics, including cervical cancer, rheumatoid arthritis, and sexual dysfunctions, has revealed significant inconsistencies in quality, with numerous videos presenting misleading or incomplete details [5–7].

Currently, there is no study in the literature assessing the quality and reliability of YouTube videos addressing RE. Misleading or inaccurate information about RE on YouTube can lead to misinformed decision-making, inappropriate self-treatment, and delayed professional care. To address this gap, we aim to evaluate the content, reliability, and quality of the most viewed YouTube videos on RE. By analyzing video content, creator credentials, and adherence to evidence-based medical standards, this study seeks to determine whether YouTube can be considered a reliable source of information for individuals affected by this condition.

## Materials and methods

### Search strategy and data collection

On YouTube, the keyword “retrograde ejaculation” was searched. The search was conducted on December 11, 2024 with an anonymous guest login, location London UK and language English. All videos (*n* = 545) resulting from the search terms were analyzed. In case of duplicate videos, only one was considered. Exclusion measures were adopted from similar previous studies as nonrelevant videos, non-English videos, or videos that had no accompanying audio [8]. After saving the search results in a playlist, 2 independent urologists (AA, CO), viewed and analyzed the videos.

### Quality and reliability assessment tools

The number of total views, views per day, likes, comments, video power index, duration on YouTube (months), and video length (seconds) were recorded for each video. Video power index was calculated as described by Erdem et al. [9].

To assess the accuracy of the videos, they were categorized into two groups: reliable and non-reliable. This approach has been utilized in multiple previous studies [7]. The detailed classification of these groups is as follows:

1. Reliable information: Videos that provided information on RE with scientifically proven accuracy, in accordance with the current recommendations of the European Urology Association (EAU) guidelines and the American Urological Association (AUA) guidelines, were classified in this group [10, 11].
2. Non-reliable information: Videos discussing the treatment of RE with scientifically unproven or incorrect information, as well as recommendations not included in the EAU and AUA guidelines, were classified in this group [10, 11]. Additionally, videos containing partially accurate or partially inaccurate information, as well as those presenting both reliable and non-reliable information, were also included in this category. Videos consisting solely of on-screen text without narration, visuals, illustrations, or any explanatory audio were classified as non-reliable, as such formats lack interactive or supportive content evaluated by DISCERN and GQS tools and are often mere transcriptions of existing written materials, limiting their educational value within a video-based platform like YouTube.

Videos were further categorized based on the source of the upload, as follows:

1. Universities, professional organizations, nonprofit entities, or physicians: This category included videos uploaded by academic institutions, professional medical organizations, nonprofit healthcare providers, or individual physicians.
2. Health information websites: Videos originating from independent health information platforms fell into this category.
3. Medical advertisements or for-profit organizations: Videos created by commercial entities promoting specific treatments or products were included here.
4. Individual users: This group encompassed videos uploaded by laypersons or non-affiliated individuals.

Additionally, videos were classified according to the identity of the speaker providing the explanation. Categories included physicians, non-physician healthcare providers, individuals appearing in the video, or an external narrator. This categorization aimed to further analyze the credibility and potential biases in the presented information.

To assess the reliability and overall quality of the videos, we utilized both the DISCERN tool and the Global Quality Score (GQS). These validated instruments help evaluate the accuracy, clarity, and educational value of health-related information.

DISCERN, initially created by Charnock et al., is a tool designed to assess the quality of health information available to patients. It consists of 15 questions evaluated on a 5-point scale [12]. Meanwhile, the GQS is a 5-point scale ranging from 1 (poor quality) to 5 (excellent quality), which considers multiple factors such as information accessibility, content accuracy and quality, and the logical flow of information within the video [13]. This comprehensive approach ensures a thorough evaluation of the educational value and usability of the videos for patients seeking information about RE.

### Statistical analysis

Statistical analyses were performed to compare the characteristics of reliable and non-reliable health information videos on YouTube. Descriptive statistics were presented as mean ± standard deviation (SD) for continuous variables and as frequencies and percentages for categorical variables. The Shapiro-Wilk test was used to assess the normality of data distribution. For comparisons between two groups (reliable vs. non-reliable information), independent samples t-tests were conducted for normally distributed continuous variables, while the Mann-Whitney U test was applied for non-normally distributed data. For comparisons involving categorical variables, the chi-square test or Fisher’s exact test was utilized, depending on the expected frequency counts. One-way analysis of variance (ANOVA) was performed to compare video characteristics among multiple subgroups based on the source of upload. Post-hoc pairwise comparisons were conducted using Tukey’s HSD test to identify significant differences between specific groups. All statistical analyses were conducted using SPSS (version 26, IBM Corp., Armonk, NY, USA).

Inter-rater agreement was evaluated using Cohen’s kappa score, while interobserver reliability was quantified through the intraclass correlation coefficient. To assess the normality of data distribution, the Kolmogorov–Smirnov test was employed. These methods ensured robust and reliable statistical analysis of the study data.

## Results

**Fig. 1:**
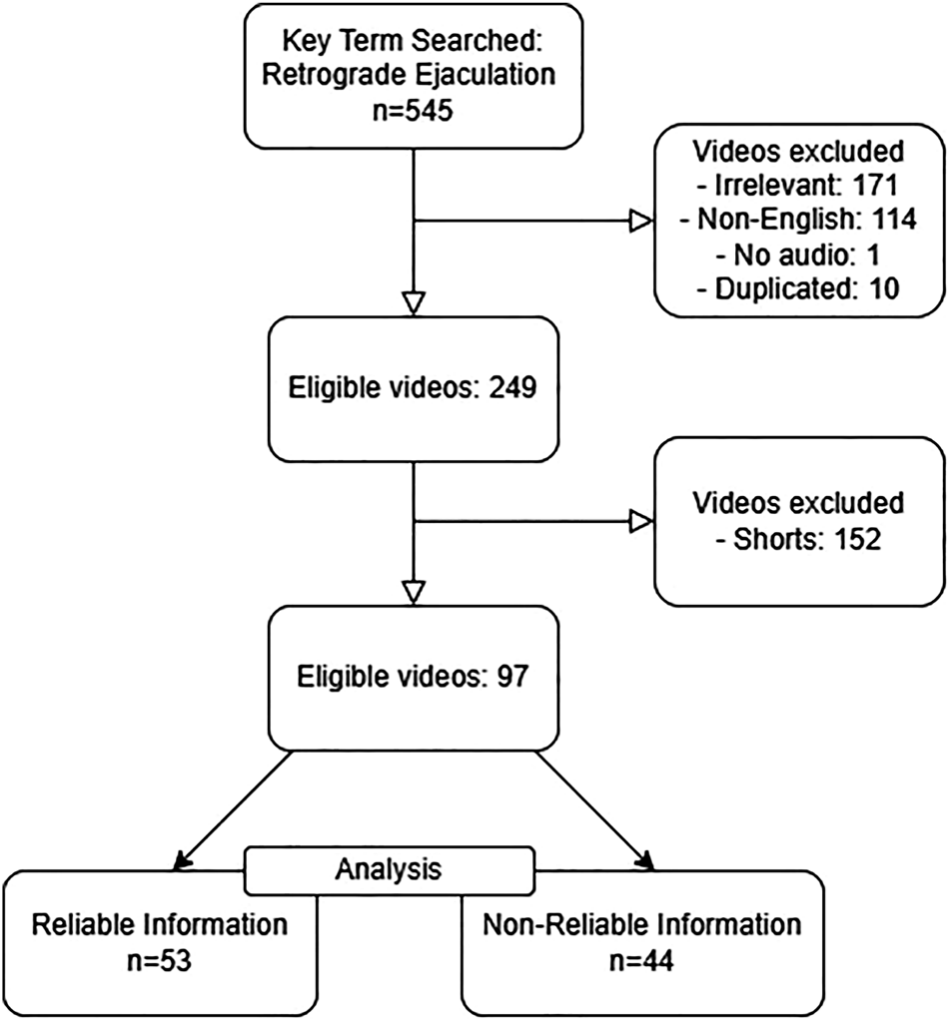
Flowchart of the study design.

A total of 545 videos were reviewed, of which 97 were included in the analysis, while 448 were excluded. The reasons for exclusion were as follows: 171 videos (31.4%) were deemed irrelevant, 114 videos (20.9%) were not in English, 1 video (0.2%) lacked audio, 10 videos (1.8%) were duplicates, and 152 videos (27.9%) were categorized as “shorts” (i.e., shortened videos) (Fig. 1).

The level of inter-rater agreement in terms of accuracy assessment of videos was positive between the two investigators (kappa coefficient = 0.794). The intraclass correlation coefficient for DISCERN score was calculated as 0.721, while it was 0.649, for GQS indicating substantial reliability. A total of 97 videos were analyzed and categorized into reliable (53 videos, 54.6%) and nonreliable (44 videos, 45.4%) information groups. Key characteristics of these videos, including viewership, duration, interaction metrics, and reliability scores, are summarized in Table 1.

**Table 1:** Comparison of characteristics between reliable and non-reliable health information on YouTube.

Characteristics	Reliable information (*N* = 53)	Non-reliable information (*N* = 44)	*P* value
Total View	39,324.85 ± 104,570.16	102,976.55 ± 429,699.32	0.948
Video Length (seconds)	699.83 ± 1484.68	554.89 ± 1401.46	0.460
Duration on YouTube (months)	37.08 ± 42.33	43.18 ± 41.46	0.219
Views per day	60.13 ± 199.43	81.08 ± 263.67	0.552
Likes	474.57 ± 1372.18	890.16 ± 2715.03	0.468
Comments	53.85 ± 135.78	75.55 ± 201.77	0.707
Video power index	60.68 ± 199.21	80.95 ± 263.66	0.533
Discern	40.79 ± 9.36	22.92 ± 5.34	***0.001***
GQS^a^	3.17 ± 0.92	1.37 ± 0.48	***0.001***
Source of upload, (n)			
-Universities/professional organizations / nonprofit physician/physician groups	26	9	***0.006***
-Stand-alone health information websites	14	10	
-Medical advertisement/for profit companies	7	11	
-Individual	6	14	
Speaker, (n)			
-Physician	29	16	***0.02***
-Non-physician health provider	3	1	
-Individual in the video	5	15	
-External voice	16	12	

- Statistical tests used: An independent samples t-test was used for Discern and GQS scores. A Mann-Whitney U test was applied for total view, video length, duration on Youtube, views per day, likes, comments, and video power index because of non-normal distribution. A Chi-square test was used for source of upload, while a Fisher’s exact test was applied for speaker.

^a^*GQS* global quality score.

No statistically significant differences were observed between reliable and nonreliable groups in terms of average views, video length, views per day, or comments (*P* = 0.948, *P* = 0.460, *P* = 0.552, *P* = 0.707 respectively). However, DISCERN scores and GQS values were significantly higher for reliable videos compared to nonreliable ones (*P* = 0.001). Reliable videos, were significantly more often uploaded by universities, professional organizations, or nonprofit physician groups when compared to non-reliable videos (*P* = 0.006).

Videos uploaded by universities, professional organizations, and nonprofit physician groups demonstrated the highest DISCERN scores (36.65 ± 11.65) and GQS values (2.77 ± 1.08), alongside an average view count of 36,854.51 ± 87,009.14 and a video length of 610.80 ± 1238.68 s. However, no significant difference was found in avarage view and video length among the subgroups (*P* = 0.339, *P* = 0.681 respectively). Videos featuring physicians as the primary speaker were associated with significantly higher reliability scores compared to those with non-physician speakers or individuals (*P* = 0.02).

In contrast, videos from medical advertisement or for-profit companies had the lowest view count (7018.00 ± 14,918.00) and shorter video lengths (302.67 ± 237.46 s), along with a lower average DISCERN scores (28.30 ± 10.02) and GQS values (1.88 ± 0.96). Videos uploaded by individuals exhibited lowest scores, with DISCERN values of 27 ± 9.28 and GQS values of 1.65 ± 0.96.

This data underscores distinct differences in reliability and quality scores based on the source of the uploaded videos, as summarized in Table 2.

**Table 2:** Comparison of video characteristics based on source and reliability of health information on YouTube.

Characteristics	Universities/professional organizations/nonprofit physician/physician groups	Stand-alone health information websites	Medical advertisement/for-profit companies	Individual	*P* value
Video number (n)	35	24	18	20	
DISCERN	36.65 ± 11.65	34.91 ± 12.90	28.30 ± 10.02	27 ± 9.28	***0.006***
GQS^a^	2.77 ± 1.08	2.70 ± 1.26	1.88 ± 0.96	1.65 ± 0.96	***<0.001***
Average views	36,854.51 ± 14,707.24	160,039.33 ± 576,295.88	7018.00 ± 14,918.00	67,880.65 ± 14,5368.34	0.339
Video length (min)	610.80 ± 1238.68	735.08 ± 1659.89	302.67 ± 237.458	851.90 ± 2050.65	0.681
Views per day	75.29 ± 235.63	65.91 ± 190.35	4.48 ± 6.24	122.84 ± 341.20	0.473
Likes	507.2 ± 1220.24	979.20 ± 3127.53	51.33 ± 108.33	1107.1 ± 2608.76	0.368
Comments	60.2 ± 129.61	83.12 ± 247.01	8.88 ± 21.30	95.8 ± 185.58	0.401
Video power index	76.23 ± 235.27	65.81 ± 190.32	4.42 ± 6.23	122.55 ± 341.24	0.472
Reliable (n)					
No	9	10	11	14	***0.005***
Yes	26	14	7	6	
Speaker (n)					
-Physician	27	8	8	2	***0.001***
-Non-physician health Provider	0	3	1	0	
-Individual in the video	0	4	3	13	
-External voice	8	9	6	5	

-One-way analysis of variance (ANOVA) was performed to compare video characteristics among multiple subgroups based on the source of upload. Post-hoc pairwise comparisons were conducted using Tukey’s HSD test to identify significant differences between specific groups.

^a^*GQS* global quality score.

## Discussion

YouTube has emerged as a major platform for disseminating health-related information, including content on RE. This study highlights the significant variability in the reliability and quality of such information. By evaluating YouTube videos on RE, we identified clear disparities in content accuracy and educational value. Reliable content, mostly from universities and professional organizations, adhered to evidence-based medical guidelines, while nonreliable videos often promoted unverified and potentially harmful information.

Reliable videos on RE predominantly originated from universities, professional organizations, or nonprofit physician groups. These videos demonstrated higher DISCERN scores and GQS values, indicating superior content quality and reliability. They often provided evidence-based guidance on RE treatments, such as alpha-adrenergic agonists (e.g., pseudoephedrine), transurethral surgical interventions, and lifestyle modifications addressing underlying conditions like diabetes or neuropathy. Conversely, nonreliable videos frequently promoted unverified remedies, such as herbal supplements and physical techniques such as tantric massages or sexual intercorse position alterations, which lacked scientific validation. Similar trends were observed in analyses of benign prostatic hyperplasia (BPH), erectile dysfunction and disorder of sexual development videos, where reliable sources emphasized established medical protocols, while nonreliable sources relied on anecdotal claims [14–16].

Interestingly, the presence of physicians as narrators did not universally ensure reliability, as some videos included promotional content for personal products. This aligns with findings from studies on varicocele, which showed that professional affiliation did not always correlate with content quality [17]. Our results underscore that the source of the upload, such as universities or professional organizations, is a more critical determinant of reliability than the narrator’s professional status.

RE can result from neurogenic causes (e.g., spinal cord injury [18], multiple sclerosis [19], autonomic neuropathy [20], surgical interventions [21], Parkinson’s disease [22], diabetes mellitus [23]) and urethral abnormalities (e.g., urethral stricture [24], ectopic ureterocele [25], congenital defects [26]). Other contributing factors include pharmacological agents (e.g., antihypertensives [1], α-1 adrenoceptor antagonists [27], psychotropic drugs [28]) and endocrine disorders [23]. Additionally, bladder neck incompetence acquired through surgical procedures such as bladder neck resection or prostatectomy, plays a significant role in the condition [29]. Evidence-based treatments, such as alpha-adrenergic medications [30] and specific surgical techniques [31], along with urinary sperm retrieval [32], prostatic massage [33], electroejaculation [34], surgical sperm extractions [35], penile vibratory stimulation [36] provide effective management options for RE based on its underlying cause. Given the individualized nature of RE treatment, decisions should consider factors such as ease of administration, invasiveness, and expected success [37]. In YouTube analyses of other sexual dysfunctions such as delayed ejaculation, phosphodiesterase type 5 inhibitors, and penile augmentation, it has also been generally concluded that the majority of videos often contain non-reliable information [38–40]. However, misinformation—particularly on platforms like YouTube—can mislead patients, delaying appropriate medical intervention or promoting harmful alternatives. Unverified treatments, such as unregulated supplements frequently advertised in unreliable videos, pose significant risks, including allergic reactions or contamination with active pharmaceutical ingredients, ultimately jeopardizing patient safety and treatment outcomes.

A lack of significant differences in viewership metrics, such as views per day and total views, between reliable and nonreliable videos reflects broader trends in YouTube health-related content. Studies on urinary incontinence and schizophrenia have shown that nonreliable videos often achieve similar, if not greater, viewer engagement due to their engaging presentation rather than their accuracy [41, 42]. This dynamic creates significant risks, as users may adopt unsafe practices, including the use of unregulated supplements or other unproven treatments for RE. The algorithmic preference for engagement over quality exacerbates the spread of misleading health content on platforms like YouTube, highlighting the need for stricter content regulation. There are already ongoing efforts in this area. An independent advisory panel, assembled by the National Academy of Medicine, analyzed and compiled data to establish key principles and attributes that could help platforms identify and potentially promote trustworthy health information sources. The discussion also highlights ethical concerns, including safeguarding free speech and personal autonomy, while stressing that social media platforms should collaborate with behavioral and public health researchers by sharing data to evaluate the impact of such policies and reinforce their own credibility [43]. However, instead of depending on partnerships between governments and large social media companies, such comprehensive research initiatives should be driven by collaborations among health organizations, ensuring that scientific accuracy and public well-being remain at the forefront. To propose a more practical solution, rather than altering the rules of a for-profit organization that prioritizes content and thought freedom, major medical organizations led by health professionals can adapt to the platform’s existing framework. Specifically, large urological associations could produce scientifically accurate, patient-friendly educational videos in multiple languages and negotiate with YouTube to ensure these videos appear at the top of relevant search results. Since YouTube already implements such prioritization for sponsored videos, a similar collaboration could be established in the interest of public health, allowing medical organizations to work with the platform to promote reliable health information.

This study is limited by its reliance on snapshot analysis, the dynamic and evolving nature of YouTube content, and the restriction to English-language videos. Furthermore, the DISCERN score, while widely used, has not been formally validated. Lastly, it is possible that we may have overlooked some reliable or unreliable videos due to the limitations of our search terms. We could have broadened the search scope by including the keywords “dry orgasm” and “dry ejaculation”; however, considering that YouTube’s search algorithm evaluates the presence of individual words, searches containing the term “dry” predominantly returned videos focused on Tantric sex, delayed ejaculation, or Eastern sexual techniques as primary results. However, we assumed that uploaders typically include the phrase “retrograde ejaculation” in either the title or keywords of their videos. As a result, any videos that might otherwise have been missed are likely to have appeared somewhere in the search results. Therefore, the impact of this limitation is expected to be minimal. Despite these limitations, this study provides valuable insights into the current landscape of YouTube videos on RE treatments, offering a foundation for further research and intervention strategies.

## Conclusion

This study demonstrates that reliable content on RE is more likely to originate from credible institutions and highlights the need for these entities to actively contribute high-quality information. Educational tools, such as certifications for verified content, could help users identify reliable sources. Moreover, YouTube must prioritize algorithmic adjustments to favor quality over engagement to mitigate the proliferation of misinformation.

## Data Availability

Additional data are available from the corresponding author on reasonable request.
